# Predisposing factors for early infection in patients with open fractures and proposal for a risk score

**DOI:** 10.1007/s10195-015-0345-z

**Published:** 2015-02-27

**Authors:** Marcos Almeida Matos, Lucynara Gomes Lima, Luiz Antonio Alcântara de Oliveira

**Affiliations:** 1 Bahian School of Medicine and Public Health, Rua da Ilha, 378, Itapuã, Salvador, Bahia 41620-620 Brazil; 2 Roberto Santos General Hospital, Salvador, Bahia Brazil; 3 Feira de Santana State University, Feira De Santana, Brazil

**Keywords:** Fracture, Infection, Treatment, Trauma, Evaluation

## Abstract

**Background:**

The primary goals of orthopedic treatment of open fractures are to prevent infection, stabilize bone injury and restore limb function. The objective of the current study was to identify risk factors associated with infection in patients suffering from open fractures, using the strength of association of these factors to propose a score that enables risk stratification in initial care.

**Materials and methods:**

A retrospective analysis was performed on 122 patients who underwent open fracture treatment. Clinical and demographic data were collected and the results were divided into two groups: those without infection and those with infection. Both groups were evaluated searching for associated factors that could lead to infection.

**Results:**

Thirty-one patients out of 122 were infected (25.4 %). Infection was significantly associated with exposure time up to 24 h (mean 30.3 h; *p* = 0.007). Fractures classified as Gustilo III had a greater chance of infection (74.2 %; *p* = 0.042), especially type IIIB (41.9 %). Fractures classified as Tscherne II and III had a greater chance of infection (48.4 and 25.8 %, respectively; *p* = 0.001).

**Conclusions:**

It was possible to show that the exposure time and the types of fracture classified as Gustilo III and Tscherne II and III are associated with the outcome of infection. It was also possible to create a risk score (IRS) for predicting infection in these types of fractures, which can be used in the initial care of the patient, with a sensitivity of 0.840, specificity of 0.544, cut-off of 6.5 and area under the curve of 0.709 (*p* = 0.002).

**Level of evidence:**

Level III.

## Introduction

Orthopedic treatment in open fractures is often performed to prevent infection, to stabilize the bone lesion and to restore limb function. The prevention of infection represents the main measure so that the other objectives may be achieved [1–3].

Post-traumatic bone infection (osteomyelitis) is a devastating event that often compromises the rehabilitation of the patient and their treatment. This infection increases the cost and duration of the treatment, causing physical and social losses, and affecting the quality of life and the functional independence of patients [4].

Early surgical debridement within 6 h and the immediate stabilization of the fracture are the most effective measures for preventing infection in the treatment of open fractures [1–3]. Even though these measures are fundamental, other clinical and environmental factors may contribute to the onset of post-traumatic osteomyelitis. The main risk factors associated with infection include trauma energy, the size of the lesion, devitalization of soft tissues, severity of the bone damage, degree of local contamination, delay in initiating treatment and the immunological status of the patient [5–7].

The identification of risk factors predictive for infection in the initial clinical evaluation of the patient with an open fracture should therefore be the crucial stage of the orthopedic treatment. The recognition of these indicators could result in more effective therapeutic measures. Thus, in the earliest instance, risk stratification could help the orthopedic surgeon to choose the best treatment for each patient.

Despite recognizing the importance of clinical and environmental risk factors in open fracture prognosis, most studies on this matter are confined to surgical aspects [8–12]. This paper seeks to identify the risk factors for infection in patients with open fractures, using the strength of the association of these factors to propose a score that may enable a risk stratification when a patient is admitted.

## Materials and methods

A retrospective study was conducted based on the records of patients who had open fractures and were treated at the Roberto Santos General Hospital (HGRS in Portuguese or RSGH in English), Salvador, Bahia, from March 2009 to December 2009.

All patients over 8 years of age with open fractures admitted through the Emergency Room of the RSGH were included. Patients coming from other units of the Public Health system of the state of Bahia who were referred to the RSGH were also included. Patients with open fractures of the axial skeleton (spine, face, skull, thorax), and those who did not remain at the unit for at least 1 day after the initial procedure, for any reason, were excluded. Patients with incomplete records were also excluded.

The RSGH is the largest public hospital in Northeastern Brazil, and a reference center for trauma surgery. The initial procedure includes filling out a standardized form for the assessment of orthopedic patients, which is attached to the records. This form is updated daily during the patient’s admission and records clinical and demographic data, as well as any occurrences related to the patient, including the presence of infection or not. This clinical form rendered this study possible and all the data used in this study was retrieved from it.

The independent variables used in the analysis were: age, sex, marital status (unmarried, married, others), origin (capital or other towns of Bahia), affected bone (upper limb and lower limb), type of accident (traffic: motorcycle, automobile, run over; gunshot wound, fall from height, direct trauma), exposure time of the fracture (time between the trauma and the therapeutic procedure), fracture classification according to Gustilo et al. [13], and classification of soft tissue lesion according to Tscherne and Oestern [14], as well as habits such as drinking and smoking. Primary treatment methods were considered as follows: cast, external fixation (all types), or internal fixation (either intra- or extra-medullary). The end result “infection” was adopted as a dependent variable.

Early infection (end result variable) was considered to be infections occurring within 2 weeks, as proposed by Willenegger [15]. The criteria to define surgical site infection in patients’ evolution followed the rules of the Center for Diseases Control and Prevention [2]. We used clinical signs and symptoms such as purulent drainage, pain, swelling, redness or fever, along with surgeon’s confirmation of the diagnosis, and also laboratory findings such as increased white cell count, raised hemosedimentation rate and C-reactive protein (CRP), and fluid cultures [15, 16]. To verify this result, the patients were evaluated at the time of admission and after a 2-week follow-up, regardless of discharge from hospital.

Data on 122 patients who met the inclusion criteria were collected. From these, the end result “infection” (dependent variable) was confirmed in 31 patients, and 91 were free of infection. The patients were thus divided into two groups: patients with and without infection.

Data were presented in tables of frequency distribution for discrete variables, and using the average and standard deviation for continuous variables. The analysis of risk factors associated with infection in both groups (with or without infection) was made using the Student *t* test for continuous variables and the chi-squared test for discrete variables. The value of *p* ≤ 0.05 was adopted as the significance level for all tests.

Considering the statistical significance found in the bivariate analysis, and with the objective of selecting variables predictive of infection, a multivariate analysis was performed. From the final model of logistic regression, the odds ratio was calculated for each variable. Thus, from the identification of variables significantly associated with infection in the bivariate and multivariate analyses, a score was created to predict the risk of infection at the time of admission, even before the initial treatment.

For the construction of the score, which was called the Infection Risk Score (IRS), relevant factors (statistic and clinical) were considered as infection predictors. Thus, three variables were included in the IRS: the Tscherne [14] and Gustilo [13] classifications, as well as the time elapsed since the fracture event. As for the exposure period, it was necessary to categorize this into the following groups: up to 12 h, from 12 to 24 h, and above 24 h. This subdivision was made to transform the time into a categorical variable, and was based on the studies of Patzakis and Wilkins [17].

For the development of the score, the exposure period of the fracture was considered as 1 for a period of up to 12 h, 2 for a period between 12 and 24 h, and 3 for a period over 24 h. For the Gustilo [13] classification, 1 was scored for type I (slight), 2 for type II (moderate), 3 for type IIIA (severe A), 4 for type IIIB (severe B) and 5 for type IIIC (severe C). For the Tscherne [14] classification, 1 was scored for type I, 2 for type II, 3 for type III and 4 for type IV. The variables were thus transformed by means of the sum of the individual scores into the final score designated IRS. These data allow the construction of the IRS, which varies from a score of 3 for the lowest infection risk to a score of 12 for the greatest infection risk.

To identify the association of the IRS with the end result of infection, the Student *t* test was used to associate the median score in both groups with the infection variable. The IRS was categorized into three levels with the object of identifying the infection risk: level I (low risk), patients with 3, 4 or 5 points in the IRS; level II (intermediate risk) for patients with 6, 7, 8 or 9 points in the IRS; and level III (high risk) for patients with 10, 11 and 12 points. In addition, a receiver operating characteristic (ROC) curve was built to show the accuracy parameters of the IRS.

## Results

The demographic characteristics of the 122 patients can be seen in Table 1. The global infection rate was 25.4 % (31 patients) and there was no statistical association between the variables studied. No association was found between infection and the anthropometric measures such as weight, age and body mass index (BMI), despite a significant difference in the height of the patients (Table 2).

**Table 1:** Sociodemographic data of patients with open fractures in a public hospital in the state of Bahia, from March to December 2009

Variable	With infection (%)	Without infection (%)	Total (%)	*p* value
*N* (%)	31 (25.4)	91 (74.6)	122	
Gender	31	91	122	0.96
Male	26 (83.9)	76 (83.5)	102 (83.6)	
Female	5 (16.1)	15 (16.5)	20 (16.4)	
Marital status	26	83	109	0.77
Unmarried	15 (57.7)	55 (66.3)	70 (64.2)	
Married	10 (38.5)	25 (30.1)	35 (32.1)	
Other	1 (3.8)	3 (3.6)	4 (3.7)	
Origin	31	90	121	0.08
City of Salvador	12 (38.7)	55 (61.1)	67 (55.4)	
Other towns	19 (61.3)	35 (38.9)	54 (44.6)	
Fracture localization	31	91	122	0.34
Lower limbs	22 (71.0)	56 (61.5)	78 (63.9)	
Upper limbs	9 (29.0)	35 (38.5)	44 (36.1)	
Type of trauma	31	91	122	0.14
Gunshot wound	5 (16.1)	29 (31.9)	34 (27.9)	
Direct trauma	10 (32.2)	23 (25.3)	33 (27.0)	
Traffic	16 (51.6)	39 (42.8)	55 (45.1)

**Table 2:** Anthropometric profile of patients with open fractures in a public hospital in the state of Bahia, from March to December, 2009

Variable	With infection	Without infection	*N* total	*p* value
Age	31.5 (±13.5)	31.7 (±14.3)	118	0.929
Weight	71.9 (±14.2)	67.9 (±14.1)	89	0.269
Height	1.76 (±0.1)	1.71 (±0.1)	84	0.036
Body mass index	23.5 (±3.0)	22.8 (±6.1)	53	0.669

For clinical conditions, all the results showed a significant association. The mean time elapsed from the trauma to the surgical treatment was 30.3 h (±19.5) for the infection group, and 21.4 h (±12.1) for the group without infection. The earliest treatment was 6 h after the trauma, and the longest length of time until treatment was 76 h after the accident. Infection had a significant association with the exposure time of the fracture. For the Gustilo [13] classification, type III fractures (74.2 %) had a greater probability of infection than other types. For the Tscherne [14] classification, lesions of type III (48.4 %) and type II (25.8 %) presented the greater risk of infection (Table 3).

**Table 3:** Clinical characteristics and treatment options of open fractures in a public hospital in the state of Bahia, from March to December, 2009

Variable	With infection	Without infection	*N* total	*p* value
Time of exposure (h)	*N* = 25	*N* = 79	94	0.007
	30.3 (±19.5)	21.4 (±12.1)		
Gustilo criteria	*N* = 31	*N* = 91	122	0.042
I	1 (3.2 %)	10 (11.0 %)	11	
II	7 (22.6 %)	39 (42.8 %)	46	
IIIA	7 (22.6 %)	20 (22.0 %)	27	
IIIB	13 (41.9 %)	19 (20.9 %)	32	
IIIC	3 (9.7 %)	3 (3.3 %)	6	
Tscherne criteria	*N* = 31	*N* = 91	122	0.001
I	6 (19.4 %)	32 (35.2 %)	38	
II	8 (25.8 %)	43 (47.2 %)	51	
III	15 (48.4 %)	15 (16.5 %)	30	
IV	2 (6.4 %)	1 (1.1 %)	3	
Treatment	*N* = 31	*N* = 91	122	0.944
Cast	42 (9.8 %)	14 (11.8 %)	56	
External fixation	31 (25.4 %)	10 (8.2 %)	41	
Internal fixation	18 (14.7 %)	7 (5.7 %)	25	

Internal fixation was the treatment choice in 25 (20.5 %) of the fractures and cast or external fixation was performed in 97 cases (79.5 %). According to the Gustilo classification, internal fixation was the method of choice in 36.45 % (*n* = 4) of type I open fractures, 15.2 % (*n* = 7) of type II, and 23.1 % (*n* = 15) of all type III. There were no statistically significant differences with regard to treatment options and infection (Table 3). There was no association between infection and treatment method when comparing only external versus internal fixation (*p* = 0.745) or cast plus external fixation versus internal fixation (*p* = 0.739).

A multivariate analysis was performed, and odds-ratio values were determined for the variables that were statistically significant in the bivariate analysis (Table 4). However, the bivariate analysis used for the IRS took into account that none of the variables were statistically significant in the multivariate analysis.

**Table 4:** Odds ratio for each variable

Variable	Estimate	95 % confidence interval
Gustilo criteria	1.328	0.731–2.411
Tscherne criteria	1.899	0.853–4.225
Exposure time	1.007	1.007–1.076

**Fig. 1:**
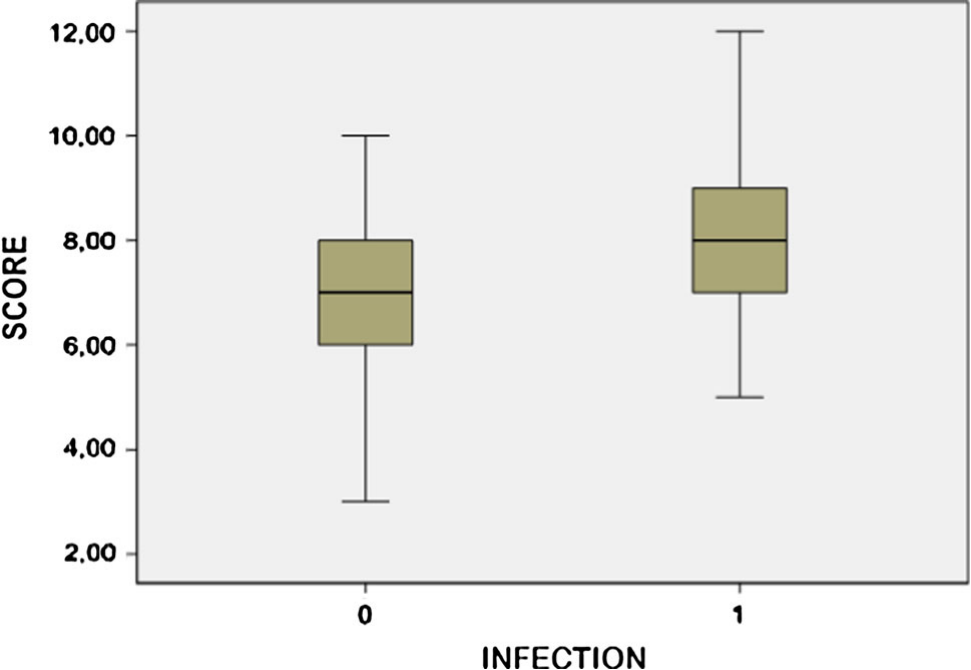
Boxplot comparing the median scores in the groups with and without infection

The IRS had a mean of 7.12 points. When we compared the means of IRS between the groups, with infection (8.24) and without infection (6.77), a statistically significant difference was observed (*p* = 0.001) (Fig. 1).

**Fig. 2:**
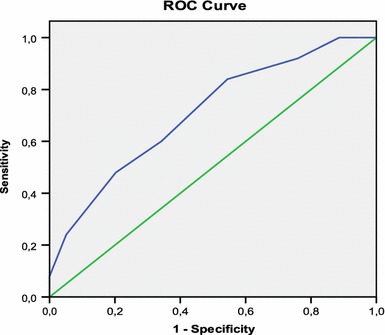
ROC curve showing the accuracy of the IRS score

The receiver operating characteristic (ROC) curve built from the IRS showed a direct correlation between the IRS and the end result “infection”; the area under the curve had an estimate of 0.709 (*p* = 0.002), a result considered satisfactory for the assessment of the association in clinical studies [18]. The accuracy of the IRS may be assessed from the characteristics of the curve at the cut-off point selected for the end result, which was 6.5. At this point, the curve presents parameters of 0.840 for sensitivity and 0.544 for specificity (Fig. 2).

## Discussion

The results of this study reveal that the clinical factors that were significantly associated with the end result “infection” were: time elapsed since the accident, type of open fracture according to Gustilo’s criteria [13], and the type of soft tissue damage according to Tscherne’s criteria [14]. These three variables were used for the construction of the IRS, resulting in a score that is able to predict the risk of infection of an open fracture at a patient’s first evaluation. The IRS had 0.84 sensitivity and 0.55 specificity at the cut-off point of 6.5. The area under the IRS ROC curve was also considered satisfactory for clinical assessment parameters (0.709) [18].

The method of treatment was not associated with the infection outcome. This fact could be explained by considering that treatment is based on infection potential and the severity of the open fracture [1, 5, 7, 8]. Therefore, the methods used were not a predictive variable. As stated in the literature, in the current sample severe open fracture or those at risk of infection were treated by external fixation in order to minimize complications [1, 5, 7, 8].

Though the greater part of the sociodemographic and anthropometric variables did not present a significant association, the assessment of these variables provided important data on the profile of the assisted population. The global infection rate was 25.4 % and in the whole sample there was a high prevalence of males (83.6 %), a mean age of 31.5 years, most were unmarried (64.2 %), lower limbs were the most involved (63.9 %) and traffic accidents represented 45.1 % of all patients in the sample. The high prevalence of alcohol (67.2 %) and tobacco (38.5 %) use should also be noted.

The sociodemographic data of the current study is in accordance with several previous reports on the same subject. Spencer et al. [9] observed a mean age of 45 years, and 40 % was traffic accident. Chua et al. [19] showed a mean age of 36.5 years, with 91.3 % of the sample being male, and traffic accidents accounting for 69 % of the individuals. Müller et al. [3] and Moore et al. [6] also found that most of the patients were male, with average ages of 35.2 and 31 years, respectively. As for the type of trauma, Moore et al. [6] found 52 % of traffic accidents and Müller et al. [3] found a prevalence of 38.4 %. Even though this type of trauma has not been identified as a factor associated with infection, it shows that the frequency of this type of high-energy trauma results in more complex open fractures, with greater chances of infection as the end result.

Bowen and Widmaier [11] show tobacco use and the patient’s immunological condition as risk factors for the development of infection. In our study, despite the high number of patients using alcohol and tobacco, this association was not confirmed. In a similar study, Pollak et al. [10] did not find any association between smoking and infection. However, the high prevalence of alcohol use reinforces the study by Arruda et al. [20], who found a strong association between accidents and the use of alcohol and illicit drugs prior to the trauma.

The overall infection rate in the current study is higher than the rates reported in previous studies. Kamat et al. [21] found an infection rate of 11.6 %, Singh et al. [22] found 14.9 %, and Spencer et al. [9] showed 14.6 % cases of infection. These reports slightly differ from the present paper. In the study by Kamat et al. [21] there were only 21.3 % of Gustilo type III fractures and all the cases were operated within a period of less than 17 h, 93 % of the Gustilo type III patients (49.5 %) in the study by Spencer et al. [9] were treated within 12 h of the injury, and 69 % of the type III fractures were treated within 6 h in the report by Singh et al. [22].

In contrast, the report by Pollak et al. [10] showed 27 % of infected fractures, which is very similar to our rate of 25.4 %. All the fractures in that study were Gustilo type III and produced by high-energy trauma, and 41.7 % of the patients were treated more than 10 h after the trauma. Our study comprised 53.3 % of Gustilo type III fractures and debridement occurred in an average of 30.3 h for the infection group, and 21.4 h for the group without infection. We believe that this higher infection rate can be explained by both severity of the wounds and a long delay in the time to treatment. Individuals coming from other towns showed greater chances of developing infection compared with those from the city of Salvador. Therefore, it is possible that some individuals, often in the most severe cases, have been referred to the RSGH because of a lack of hospital units or adequate therapeutic means.

The classifications by Gustilo [13] and Tscherne [14] have been shown in this study to be important predictive factors for infection. This agrees with most papers using Gustilo’s classification, but there are also a few studies that evaluate infection predictors using Tscherne’s classification. Gustilo et al. [23] showed in their paper a 0 % infection rate for type I fractures, 2.5 % for type II, 13.7 % for type IIIA, 5 % for type IIIB and 44.4 % for type IIIC. In comparison, Müller et al. [3] demonstrated an infection rate of 68.8 % for Gustilo type III for an exposure time greater than 6 h. Recently, Chua et al. [19] have shown a rate of 8.5 % of infection in type I, 9.4 % in type II, 21.8 % in type IIIA and 44.6 % in types IIIB and IIIC. The lesions more strongly associated with infection found in our study, according to Tscherne’s soft tissue lesion classification, were type II and III. These findings also agree with Müller et al. [3], who found a high rate of infection in Tscherne type III and IV lesions.

Although there is no consensus in the orthopedic literature regarding the correlation between time and infection, there is evidence of its strength. Spencer et al. [9], Kamat et al. [21] and Singh et al. [22] did not find an association between infection and time to first debridement. Conversely, in our study, this variable was statistically significant, and thus an important predictive factor for infection in open fractures. Kindsfater and Jonassen [24] made a comparative study of tibial fractures grades II and III in which there were different statistical results in relation to osteomyelitis in the groups operated earlier and later than 5 h after the trauma (7 and 38 %, respectively). In the study by Pollak et al. [10], infection was not associated with the time delay from injury to debridement, but time from injury to admission was a predictor of infection, considering time as a continuous variable. In the same study, considering time as a categorical variable, there was a significant chance of infection when time to admission was longer than 2 h (5.4 times more likely to have development of infection) and the risk of infection was significantly higher when patients were transferred to the trauma center after 11 h.

All these previous studies presented time as a categorical variable divided into multiples of 5 or 6 h. However, only a few fractures were treated after 12 h: for instance, just eight (7 %) in the study by Spencer et al. [9]. In our study, time was also used as a categorical variable. Thus, we used intervals of 12 h, similar to the model adopted by Patzakis and Wilkins [17]. This division was used because only nine fractures (7.3 %) were treated in less than 6 h after the time of exposure; the majority were treated after 12 h. Therefore, we believe that time from injury to admission (or debridement) as a categorical variable is a significant predictor when the delay is more than 12 h, and that may conform with and elucidate some of the previous findings in the orthopedic literature.

Though orthopedists agree that prevention of infection is a crucial matter in the treatment of open fractures, few studies have been dedicated to the assessment of predictive factors in these cases. Our study analyzed factors associated with infection and those factors with a stronger association were combined to build a score designated IRS. This score has been shown to be satisfactory in its objectives and especially adequate regarding its sensitivity for infection (84 %). No similar scores have been found in the literature, though much emphasis has been given to individual variables associated with infection, especially the time elapsed between the accident and actual treatment, and the severity of the lesion according to Gustilo [13].

The basic utility of the IRS is to create a useful tool for predicting the risk of infection in open fractures at the moment of the patient’s admission to the Emergency Room, keeping in mind that all variables used for the IRS are collected at the initial clinical assessment, and thus post-operative or laboratory results are not necessary for this tool. The IRS could, therefore, guide an orthopedic surgeon in the first surgical approach, which would be as cautious as the infection risk requires. Factors such as debridement extension, primary closure of the lesion, type and time of antibiotics, and type of fracture fixation could be decided based on the IRS. Post-operative therapeutics, nursing care and patient rehabilitation could also be provided according to the IRS score.

This paper was developed using data from patient’s records which were not always complete, thus preventing a complete analysis that could have complemented the study. In addition, some statistical sub-analyses could have suffered distortions as the sample size was calculated specifically for the infection end result. Time was also a limitation factor in constructing the score, because exposure time was categorized in a subjective way, trying to categorize time as homogeneously as possible.

This paper furnishes the literature with several original contributions on the theme. Our data has reinforced the association between infection and time of exposure and lesion severity variables in open fractures. Gustilo’s classification has already been related to infection several times in the literature, but our study represents one of the few in which Tscherne’s classification has been used, thus supporting its strongest association among all factors. Creating a risk score (IRS) to predict infection with 0.840 sensitivity and 0.544 specificity which may be used at the initial presentation of the patient may also constitute an important contribution which could be used in future studies bearing in mind its validation.
